# Environmental and Anthropogenic Factors Shape the Skin Bacterial Communities of a Semi-Arid Amphibian Species

**DOI:** 10.1007/s00248-022-02130-5

**Published:** 2022-11-29

**Authors:** K. A. Bates, J. Friesen, A. Loyau, H. Butler, V. T. Vredenburg, J. Laufer, A. Chatzinotas, D. S. Schmeller

**Affiliations:** 1 Department of Zoology, University of Oxford, Oxford, UK; 2 Centre for Environmental Biotechnology, Helmholtz Centre for Environmental Research–UFZ, Leipzig, Germany; 3 Department of Experimental Limnology, Leibniz-Institute of Freshwater Ecology and Inland Fisheries (IGB), Stechlin, Germany; 4 Laboratoire Écologie Fonctionnelle et Environnement, Université de Toulouse, INPT, UPS, Toulouse, France; 5 Department of Biology, San Francisco State University, San Francisco, CA USA; 6 Department of Environmental Microbiology, Helmholtz Centre for Environmental Research–UFZ, Leipzig, Germany; 7 Institute of Biology, Leipzig University, Leipzig, Germany; 8 German Centre for Integrative Biodiversity Research (iDiv), Halle-Jena-Leipzig, Leipzig, Germany

**Keywords:** Ranavirus, Amphibian, Skin microbiome, Habitat disturbance, *Batrachochytrium dendrobatidis*, Toad

## Abstract

The amphibian skin microbiome is important in maintaining host health, but is vulnerable to perturbation from changes in biotic and abiotic conditions. Anthropogenic habitat disturbance and emerging infectious diseases are both potential disrupters of the skin microbiome, in addition to being major drivers of amphibian decline globally. We investigated how host environment (hydrology, habitat disturbance), pathogen presence, and host biology (life stage) impact the skin microbiome of wild Dhofar toads (*Duttaphrynus dhufarensis*) in Oman. We detected ranavirus (but not *Batrachochytrium dendrobatidis*) across all sampling sites, constituting the first report of this pathogen in Oman, with reduced prevalence in disturbed sites. We show that skin microbiome beta diversity is driven by host life stage, water source, and habitat disturbance, but not ranavirus infection. Finally, although trends in bacterial diversity and differential abundance were evident in disturbed versus undisturbed sites, bacterial co-occurrence patterns determined through network analyses revealed high site specificity. Our results therefore provide support for amphibian skin microbiome diversity and taxa abundance being associated with habitat disturbance, with bacterial co-occurrence (and likely broader aspects of microbial community ecology) being largely site specific.

**Supplementary Information:**

The online version contains supplementary material available at 10.1007/s00248-022-02130-5.

## Introduction 

The skin microbiome of vertebrates such as amphibians has gained increasing recognition for its importance in broader aspects of host health including maintaining surface integrity [1], educating the immune system [2–4], and interacting with invading pathogens [5, 6]. As the primary interface between the host and its environment, the skin and its diverse microbiota are influenced by myriad variables including host factors [7], diet [8], the environment [9–12], and microbial invaders [11, 13–18]. Changes to skin microbial community structure driven by perturbations to any one of these factors (or their interaction) can therefore have profound consequences for host health [19].

Major threats to amphibians include habitat disturbance and emerging infectious diseases, which have contributed to the global decline of this vertebrate group, with over 40% of species at risk of extinction [20–25]. Anthropogenic disturbances such as environmental contamination or habitat disruption not only provide dispersal opportunities for environmental microbes [26], but also are major stressors on amphibians that can drive perturbations of the skin microbiome [12, 27–30] as well as influence disease epidemiology [31, 32]. For example, environmental contamination through run-off from livestock farming can promote the growth of faecal coliform bacteria, subsequently altering the skin microbiome of resident amphibians by colonisation of pathogen-facilitating bacteria, that in turn have been linked with increased prevalence of the fungal pathogen *Batrachochytrium dendrobatidis (Bd)* [30]. Conversely, another study found that habitat disturbance increased skin microbiome dispersion, but was linked to lower *Bd* prevalence [33]. Deciphering the impacts of biotic and abiotic variables (including habitat disturbance) on the microbiome and disease ecology is therefore integral to our understanding of amphibian health and conservation [34]. The sensitivity of amphibians to environmental change also makes them useful ecological indicators [35, 36]. Further, the skin microbiome’s close coupling with the environment lends itself as a novel diagnostic target, providing a non-invasive snapshot of host health, as well as responding to subtle disturbances to the wider ecosystem that might otherwise go unnoticed [37].

We investigated how host biology (life stage), environmental parameters (local hydrology, habitat disturbance), and pathogen presence shape the skin microbiome of Dhofar toads (*Duttaphrynus dhufarensis*) in the Dhofar Mountains of Oman. This toad is one of two amphibian species present in Oman and the only one present in the south. To investigate disease epidemiology, we surveyed wild populations of *D. dhufarensis* for *Batrachochytrium dendrobatidis* (*Bd*) and ranavirus. Whilst these pathogens have caused severe declines in wild amphibians [25, 38–40], their presence on the Arabian peninsula has been overlooked [41, 42], with no prior reports of either pathogen in Oman. We assigned major water sources to the sampling sites, in addition to estimating anthropogenic disturbance by quantifying roads and built-up area using OpenStreetMap (OSM) [43] and World Settlement Footprint (WSM) [44] data. Importantly, prior studies have demonstrated that water source and habitat disturbance can influence the amphibian skin microbiome, yet much remains to be understood regarding their relative importance in shaping the microbiome and disease when considered together [12, 27, 45, 46].

We found that ranavirus but not *Bd* is prevalent across wild *D. dhufarensis* locations, with reduced prevalence in disturbed sites, and representing the first detection of ranavirus in Oman. We further demonstrate that host life stage exhibits significantly higher alpha diversity in adults compared to larvae. Among post-metamorphic animals, habitat disturbance, water source, and ranavirus infection were not associated with bacterial alpha diversity. Conversely, we found that dermal bacterial beta diversity was strongly structured by water source, habitat disturbance, and life stage, but not ranavirus presence. Despite differences in beta diversity and taxa abundance between disturbed and undisturbed sites, we show that bacterial co-occurrence as measured through correlation networks was largely site specific, with very few shared bacterial associations across sites. Our results therefore progress our understanding of the key parameters driving dermal bacterial community composition in an amphibian species from a semi-arid habitat, and further raise the potential of the amphibian skin microbiome as a novel tool in measuring wider ecosystem health.

## Methods

### Field Sampling

**Fig. 1:**
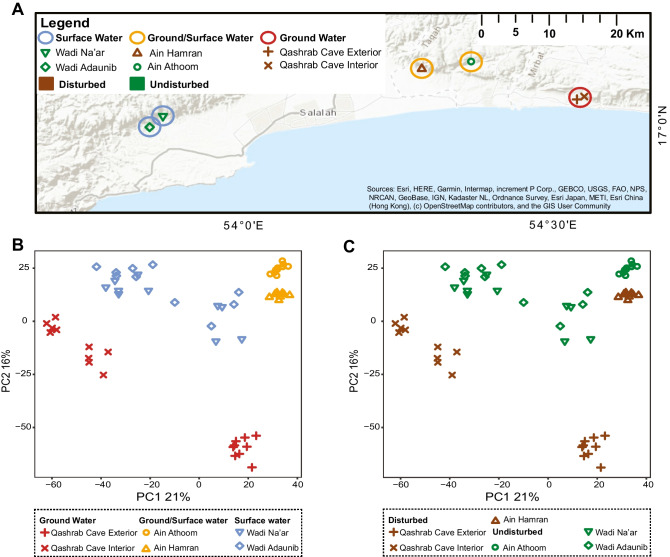
**A** Map of sites. Skin bacterial beta diversity colured by **B** water source and **C** disturbance

Individuals of the Dhofar toad (*Duttaphrynus dhufarensis*) were sampled from six sites in the Dhofar Mountains, Oman, in August 2017 (Fig. 1A). The sampling sites were situated in ephemeral water sources or wadis (Wadi Na’ar, Wadi Adaunib), groundwater spring sites (Ain Hamran Ain Athoom), in an underground cave (Qashrab Cave Interior), and directly outside the entrance to the underground cave (Qashrab Cave Exterior). Our sampling primarily focused on post-metamorphic animals (adults, both males and females, who gathered for reproduction, snout to vent length range of 5.5–7 cm), with exception of Wadi Na’ar where larvae (approximately 1–2 days old), were also sampled to allow for comparison of life stage effects in the microbiome. To sample the microbiome, post-metamorphic and larval *D. dhufarensis* were captured (either with a net or by hand with nitrile gloves) and swabbed over the head, tail, and ventral abdomen using single sterile MW100 rayon tipped dry swabs (MWE Medical Wire, Corsham, UK). In post-metamorphic animals, *Batrachochytrium dendrobatidis* (*Bd*) and ranavirus (RV) were sampled by rolling the swab tip over the head, tail, ventral abdomen, and cloaca. In larvae, *Bd* was sampled from the keratinised mouthparts. Environmental water microbiome was sampled by filtering 250 mL of environmental water on a sterile Millipore cellulose acetate filter (0.22 µm) with a hand vacuum pump. We strictly applied international standards on hygiene protocol to prevent dissemination of pathogens when working with amphibians [47].

### Hydrological Profiling

The six sampling sites are fed by precipitation and river runoff, as well as by groundwater springs. We classified the two wadi sampling sites (Wadi Na'ar and Wadi Adaunib) that are mostly supplied by surface water as “Surface Water” fed and Ain Hamran and Ain Athoom as “Ground/Surface water” fed, whilst we classified Qashrab Cave Exterior and Interior as being “Groundwater” fed.

### Anthropogenic Disturbance

The Dhofar Mountains are home to the unique semi-arid Dhofar cloud forest [48]. Due to anthropogenic pressures such as livestock grazing and infrastructure developments, the cloud forest now has a patchy distribution throughout the Dhofar mountain range [49]. Road network data from OpenStreetMap (OSM) used to calculate road density (road length [m] per km^2^) were extracted for a 2 km radius around each sampling site. For the same 2 km radius, built-up area was extracted from the World Settlement Footprint (WSF 2019) dataset [44]. OSM data were downloaded through GeoFabrik (http://download.geofabrik.de/asia/gcc-states.html) in August 2019. We grouped sites as disturbed or undisturbed based on high or low road density and built-up area, respectively (Table 1). Sites classified as undisturbed were Wadi Na’ar, Wadi Adaunib, and Ain Athoom. The disturbed sites included Qashrab Cave Exterior, Qashrab Cave Interior, and Ain Hamran.

**Table 1:** Summary of sites

Site	Water source	Buildings (m^2^) within 2 km	Road length (m) within 2 km	Disturbance category	Number of individuals sampled	Number of infected individuals	Infection prevalence (%)	Average infection intensity (MCP)	Range (MCP)
Wadi Na’ar (Adults)	Surface	0	1316	Undisturbed	10	5	50.00	8.64	0.00–24.20
Wadi Na’ar (Larvae)	Surface	0	1316	Undisturbed	10	2	20.00	8.00	0.00–15.98
Ain Athoom (Adults)	Ground/surface water	400	2835	Undisturbed	10	4	40.00	219.30	0.00–273.93
Wadi Adaunib (Adults)	Surface water	0	3226	Undisturbed	10	6	60.00	1925.66	0.00–10,731.68
Qashrab Cave Exterior (Adults)	Groundwater	22,000	18,751	Disturbed	9	3	33.33	21.10	0.00–45.86
Qashrab Cave Interior (Adults)	Groundwater	22,000	18,751	Disturbed	10	3	30.00	106.50	0.00–110.36
Ain Hamran (Adults)	Groundwater/surface water	2600	6445	Disturbed	10	1	10.00	173.66	0.00–173.66

### Pathogen Detection

Genomic DNA from *Bd* swabs was extracted using a bead-beating protocol [50] before being diluted 1/10 in distilled water for subsequent qPCR amplification. Samples were run along with negative controls (H_2_0, TE buffer) and positive controls at dilutions of 100, 10, 1, and 0.1 genomic equivalents (GE). The raw genomic equivalents output was multiplied by 150 to account for the dilution factor of 1:150, giving a relative measure in terms of genomic equivalents (GE). The presence of ranavirus was assayed by real-time PCR [51] with the inclusion of a negative control (H_2_O, TE buffer) and positive controls at dilutions of 3, 30, 300, and 3000 major capsid protein (MCP) gene equivalents. Raw MCP output was multiplied by 250 to account for the dilution factor of 1:250. A sample was considered positive if the amplification curve was sigmoidal with an MCP/GE value greater than zero.

### 16S rRNA Gene Sequencing

Skin bacterial communities and water samples were analysed using 16S rRNA gene amplicon sequencing. DNA was extracted using the Macherey Nagel Nucleospin soil kit (Macherey–Nagel GmbH and Co. KG, Düren, Germany) using the established protocol, and the hypervariable V3-V4 region of the bacterial 16S gene was amplified in triplicate using primers with overhang adaptors. Each 25 µL reaction consisted of 12.5 µL KAPA HiFi HotStart ReadyMix (KAPA Biosystems, Wilmington, MA, USA), 5 µL forward and reverse primers (1 M), and 2.5 µL template. PCR conditions were 95 °C for 180 s, followed by 25 cycles of 95 °C for 30 s, 60 °C for 15 s, 72 °C for 45 s, and a final extension of 72 °C for 120 s. PCR replicates from each sample were pooled and purified using solid phase reversible immobilisation (SPRI) beads (Agencourt AMPure XT, Agencourt Bioscience Corporation, Beverly, MA, USA). All samples and negative controls (either swabs or filters going through the same extraction procedures as the samples) were visualised using gel electrophoresis. Dual indices, provided by the Illumina Nextera Index Kit (Illumina, Inc., San Diego, CA, USA), were attached to the purified amplicons using PCR. Each 25 µL reaction consisted of 12.5 µL Kapa HiFi HotStart ReadyMix, 1 µL forward and reverse primers (1 µM), 0.5 µL BSA, 5 µL PCR-grade water, and 5 µL template. PCR conditions were 95 °C for 180 s, followed by 10 cycles of 95 °C for 30 s, 55 °C for 30 s, 72 °C for 30 s, and a final extension of 72 °C for 300 s. PCR product was purified and visualised as described above. DNA concentrations were quantified using Qubit fluorometric quantification (Life Technologies, California, USA), and samples were diluted and pooled at equimolar concentrations. Sequencing was performed on an Illumina MiSeq using a MiSeq Reagent Kit v3 (600 cycle) (Illumina, Inc., San Diego, CA, USA).

### Microbiome Sequence Processing and Analysis

We performed sequence processing in DADA2 [52] v1.16.0 using the default pipeline to infer amplicon sequence variants (ASVs). Primers were removed, and reads were trimmed. Sequence data was quality filtered by trimming reads at the first appearance of a base with a quality score of two or lower, excluding reads with non-assigned bases, and removing reads with an expected error rate higher than two. Reads matching the PhiX sequencing standard genome were also removed. Since data was generated across two sequencing runs, we learned error rates individually for each run before merging data for chimaera removal. We assigned taxonomy using the Silva database version 138 [53]. A phyloseq object [54] was created for further processing and analysis. Contaminant sequences were removed using the decontam package v.1.6.0 [55]. ASVs taxonomically assigned as chloroplast along with unclassified phyla were removed leaving a total of 29,223 ASVs (sample range: 13,302–107,992 reads).

### Statistical Analysis

#### Analysis of Infection Data

We examined whether there was an association between ranavirus infection prevalence and habitat disturbance, water source, or skin microbiome Shannon diversity (site average) using a generalised linear model with a binomially distributed response variable. A starting model containing all predictors was simplified by backward step elimination to obtain a minimum adequate model based on Akaike information criterion (AIC). Significance of predictors in the final model was calculated using a likelihood ratio test. *Bd* was not detected in our sampling and therefore not included in any models.

#### Alpha Diversity

For analysis of alpha diversity, to mitigate the effects of uneven sampling [56], microbiome samples were rarefied to 13,302 reads (post-metamorphic animals) and 32,804 (life stage analysis) corresponding to the depth of the lowest read samples in each case. To investigate differences in the microbiome associated with life stage, we focussed on Wadi Na’ar since we sampled both larvae and post-metamorphic animals from this site. We compared bacterial Shannon diversity between life stages using a *t*-test. To examine whether bacterial Shannon diversity in post-metamorphic animals was predicted by disturbance, ranavirus infection intensity (log_10_ + 1 transformed), or water source, we performed a linear mixed effects model using the lme4 package [57] with site included as a random intercept term to account for baseline differences among locations. Due to the relatively small sample sizes of each site, significance of fixed effects were assessed using the Kenward Rogers method in afex [58] to reduce type I error rate [59].

#### Beta Diversity and Differential Abundance Analysis

To analyse beta diversity and determine differentially abundant bacterial taxa, we considered the compositional nature of microbiome data [60]. First, to reduce the sparsity (abundance of zeros) in our datasets, we filtered the unrarefied microbiome data to include taxa with a relative abundance > 0.01%. We centred log ratio (CLR) transformed ASV abundances using the microbiome package in R [61].

We performed permutational multivariate analysis of variance (PERMANOVA) on the Euclidean distance matrix of bacterial data using the *adonis* function in the vegan package [62] with 10,000 permutations to examine if beta diversity differed by disturbance, water source, and site in post-metamorphic animals. Since the output of *adonis* is dependent on the order of explanatory variables (a terms explanatory power depends on what is fitted before it), we first fitted a model with only site as a predictor to estimate the overall variance that it explained. We subsequently performed a multivariate model including disturbance, water source, and site as predictors. We also performed *adonis* with ranavirus presence/absence as the the predictor and permutations constrained within locations using the strata argument. To examine whether microbiome dispersion differed based on variables of interest in post-metamorphic animals, we calculated the within-site divergence metric of beta diversity based on Euclidean distance using the microbiome package [61]. Log 10 transformed divergence values were then fitted as a response variable in a Gaussian mixed effects model with disturbance and ranavirus presence/absence as fixed effects and site ID as a random effect. We assessed whether ranavirus load was associated with beta diversity using partial Mantel tests to correlate a distance matrix of log_10_ + 1 ranavirus load with Euclidean distances of bacterial composition, whilst also accounting for geographic distance between sites. PERMANOVA and beta dispersion were also performed with life stage as a predictor.

To identify taxa driving differences in beta diversity based on disturbance or life stage, we used ALDEx2 on the top 0.01% of ASVs [63]. To compare compositional similarities between sites and categories of interest (e.g. host and environment or life stage), we calculated the number of ASVs that were shared among sites, using the top 0.01% of untransformed ASVs, and visualised the results using UpSetR [64].

#### Network Analysis

We performed network analysis on the top 0.01% of ASVs using the R package NetCoMi [65]. Using CLR-transformed ASV data, we calculated Spearman correlations between taxa and visualised interactions with *ρ* > 0.7 (strong positive interactions) or *ρ* < − 0.7 (strong negative interactions). Spearman’s correlation was selected as it can consider non-linear relationships between taxa (a common feature of microbial communities), which many other common co-occurrence methods do not [66]. Network properties including global network parameters and node topologies were estimated using the *netAnalyze* function. We also compared keystone/hub taxa between sites/life stage, which were identified based on high closeness centrality and node degree values (greater than the 90% quantile of the fitted log-normal distribution of all nodes) [67, 68]. UpsetR [64] was used to calculate common edges among site networks.

## Results

### Pathogen Presence

Ranavirus was present at all sites, with great prevalence in Wadi Adaunib (Table 1). The average ranavirus infection intensity across all post-metamorphic individuals sampled was 220.86 MCP, with an average site prevalence of 37% across post-metamorphic animals and 20% in the single larvale site. The best binomial GLM model (lowest AIC) included disturbance only as a predictor and showed that undisturbed sites had significantly higher ranavirus prevalence than disturbed sites (*χ*^2^_1_ = 4.292, *p* = 0.038). *Bd* was not detected in any animals sampled.

### Host Environment and Anthropogenic Disturbance Shape the Adult Microbiome

Bacterial alpha diversity in post-metamorphic animals did not differ significantly based on disturbance, water source, or ranavirus infection load (LMM, *p* > 0.05). Host location was significant in explaining variance in beta diversity in both univariate (PERMANOVA, pseudo-*F*_(5,53)_ = 12.484, *R*^2^ = 0.541, *p* < 0.001) and multivariate (PERMANOVA, pseudo-*F*_(2,53)_ = 11.192, *R*^2^ = 0.194, *p* < 0.001) models. Anthropogenic disturbance and water source were also significant in explaining beta diversity (PERMANOVA, disturbance: pseudo-*F*_(1,53)_ = 11.284, *R*^2^ = 0.098, *p* < 0.001; water source: pseudo-*F*_(2,53)_ = 14.376, *R*^2^ = 0.249, *p* < 0.001, Fig. 1B and C). Ranavirus presence did not drive significant differences in beta diversity (PERMANOVA, ranavirus presence: pseudo-*F*_(1,57) _= 1.200, *R*^2^ = 0.021, *p* > 0.05, SI Fig. 1). Beta diversity divergence was not significantly associated with disturbance or ranavirus presence (LMM, *p* < 0.05). We found no significant correlation between ranavirus infection load and bacterial beta diversity (partial Mantel test, *p* > 0.05).

Differential abundance analysis using ALDEx2 based on habitat disturbance yielded a single ASV belonging to the *Chroococcidiopsis* genus which was associated with disturbance (SI Table 1, SI Fig. 2). Across all locations, a small subset of ASVs were common to both environmental samples and post-metamorphic animals (range: 7–28%) (SI Fig. 3). Analysis of ASVs among post-metamorphic animals revealed that Qashrab Cave Interior, Wadi Adaunib, and Wadi Na’ar had the highest number of common ASVs (80), with 56 ASVs common to all locations (SI Fig. 4). No ASVs were unique to all three undisturbed locations; however, 15 ASVs were present across all disturbed locations but were not detected in the undisturbed locations (SI Fig. 4).

**Fig. 2:**
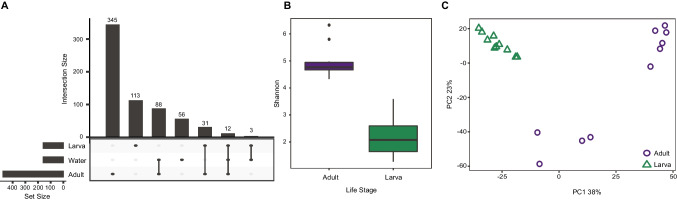
**A** Shared skin bacterial ASVs between life stages and environment **B** bacterial Shannon diversity in adults and larvae **C** PCA of bacterial beta diversity based on host life stage

We inferred bacterial associations on the amphibian skin based on Spearman correlations (SI Fig. 5). Despite heterogeneity in the data in terms of shared ASVs among locations and differences in community composition, we found that networks for post-metamorphic animals from each location were broadly similar in terms of the number of nodes (range: 399–575), edges (range: 5198–22,357), and topological features (Table 2). Networks across all post-metamorphic locations were dominated by positive correlations between bacterial taxa (% negative edges range: 8.66–25.85%) (Table 2). Networks for each location were all relatively poorly dense (0.06547–0.20459 edge density) and showed comparable clustering coefficients (0.49756–0.71159). Modularity ranged between 0.24890 and 0.51784 (Table 2).

**Table 2:** Bacterial network properties for post-metamorphic animals from each site

Water source	Disturbance	Lake	No. Node	No. Edges	% negative edges	Edge density	Network modularity	Number of modules	Clustering co-efficient	Average path length	Hubs
S	Undisturbed	Wadi Na’ar	476	16,465	15.16	0.14564	0.38626	6	0.66772	1.04160	0
GW/S	Undisturbed	Ain Athoom	399	5,198	8.66	0.06547	0.39612	8	0.49756	1.18649	39
S	Undisturbed	Wadi Adaunib	468	22,357	25.85	0.20459	0.24890	5	0.71159	1.09004	0
GW	Disturbed	Qashrab Cave Interior	575	11,840	15.49	0.07175	0.51784	7	0.57185	1.09353	19
GW	Disturbed	Qashrab Cave Exterior	492	9,238	14.64	0.07648	0.38295	8	0.51848	1.02045	56
GW/S	Disturbed	Ain Hamran	437	9,942	8.95	0.10436	0.34910	5	0.57261	1.10838	14

All networks were similar in taxonomic composition and were dominated by four phyla (Proteobacteria, Firmicutes, Cyanobacteria, and Bacteroidota), with Proteobacteria or Firmicutes contributing to the largest number of nodes at each site. Bacterial keystone/hub taxa that are likely to be of functional importance within the microbial communities differed between sites, with the number of hubs per location ranging from 0 (Wadi Na’ar and Wadi Adaunib) to 56 (Qashrab Cave Exterior) (SI Table 2). Hub taxa were predominantly site specific, with the greatest number of hubs common to both Ain Athoom and Qashrab Cave Exterior (six), followed by Qashrab Cave Exterior and Ain Hamran and Qashrab Cave Interior and Ain Athoom each having two hubs in common. A single hub taxon (ASV976_*Sphingomonas*) was common to Ain Hamran and Ain Athoom (SI Fig. 6). The majority of edges were unique to each site, with 39 edges shared uniquely among undisturbed sites, 12 edges shared uniquely among disturbed sites, and zero edges common to all six sites (SI Fig. 7).

### Microbiome is Structured by Life Stage

We identified 43 ASVs shared between adults and larvae in Wadi Na’ar, 15 ASVs shared between larvae and pond water, and 100 ASVs shared between adults and pond water (Fig. 2A). A total of 12 ASVs were common to pond water and both host life stages. Microbial alpha diversity was significantly higher in adults compared to larvae (*t*_(17.70)_ = 9.451, *p* < 0.001, Fig. 2B). Beta diversity differed significantly according to life stage (PERMANOVA pseudo-*F*_(1,18)_ = 8.767, *R*^2^ = 0.328, *p* < 0.001, Fig. 2C). Beta dispersion significantly differed by life stage, with higher dispersion in adults than larvae (*F*_(1,18)_ = 18.375, *p* < 0.001). We identified 68 differentially abundant ASVs between life stages that were dominated by members of the Gammaproteobacteria, Cyanobacteria, Clostridia, Bacteroidia, and Alphaproteobacteria (SI Table 3).

Global network properties for adults and larvae at Wadi Na’ar were similar across all topological features measured (SI Fig. 8, SI Table 4). Bacterial co-occurrence however differed greatly, with only 0.1% of edges common to both adult and larval networks. In addition, whilst no hub taxa were identified for the adult network, a total of 11 hubs were present in the larval network (SI Table 5).

## Discussion

As human activity expands, native species are increasingly exposed to new pressures such as habitat change/loss and emerging pathogens [69–71]. Identifying the major factors that shape the amphibian microbiome across host life stages is therefore critical in enhancing our understanding of host microbial ecology, enabling better monitoring of amphibian health, as well as potentially detecting the early stages of ecosystem distress.

Here, we investigated how environmental factors (habitat disturbance, local hydrology), pathogen presence, and host life stage impact the skin microbiome of the Dhofar toad. To our knowledge, we provide the first survey of ranavirus in Oman, demonstrating its presence across all sites sampled, with reduced prevalence in disturbed sites. This finding is contrary to those of other ranavirus studies, which have found ranavirus spread and prevalence to be coupled with human activity and urban environments [72–74]. The limited ranavirus prevalence in disturbed sites observed here is, however, consistent with other amphibian disease systems, such as *Bd* [33, 75]. Given that ranavirus has broad host range in ectothermic vertebrates [76] and its prevalence has been shown to increase with amphibian and fish diversity [74], a potential driver for reduced disease prevalence in disturbed sites may therefore be fewer potential ranavirus carriers (in this case fish owing to already low endemic amphibian diversity). Alternatively, if disturbed locations have reduced predator abundance (therefore minimising host stress responses) [74, 77], infection may be minimised. Habitat disturbance may also alter the local microclimate [78, 79], and this has potential to limit pathogen proliferation [80]. Determining what underpins patterns of prevalence in disturbed and undisturbed sites will ultimately require further studies incorporating additional biotic and abiotic factors for each site, such as species diversity, temperature, and humidity.

We found that amphibian skin bacterial alpha diversity was not significantly linked to habitat disturbance, water source, or ranavirus infection, supporting results of prior studies [18, 27, 28]. We did however discover that site, water source, and habitat disturbance, but not ranavirus infection, were important predictors of post-metamorphic amphibian skin microbial community structure and composition. The large proportion of variance in beta diversity attributed to site and water source suggests that habitat-specific environmental features are the major determinants of host skin bacterial community structure. Despite evidence of site and water source driving beta diversity, we found that the host-associated microbiome is distinct to that of the environment as indicated by the relatively low percentage of ASVs (7–28%) that were common to post-metamorphic animals and environmental samples. Consistent with prior studies [16], this finding may reflect a low abundance of host-associated taxa in the environment that were not detected from sequencing or were excluded during bioinformatic processing. Alternatively, the strong signature of site and water source in shaping microbiome community composition may be due to habitat-specific selection pressures that drive proliferation of different bacterial taxa. The variability in host microbiome across water sources is not surprising given the documented abiotic differences between ground and surface water and the subsequent effects on the water microbiome [81]. In particular, groundwater generally has a longer residence time, a lower concentration of dissolved organic matter, and differs in light and oxygen levels compared to surface water [81–83]. Microbial community composition also varies between ground and surface water environments, as well as modes of microbial metabolism, with heterotrophy and chemoautotrophy associated with groundwater, and photoautotrophy and heterotrophy occurring in surface water [81, 83]. Exploration of the specific hydrological parameters for each water source and how these influence the water microbiome may therefore provide a mechanistic link between abiotic factors, the environmental microbiome, and how these interact to shape the host skin microbiome. This line of research may be especially valuable when considering the host environment in species re-introductions or restoration ecology. That only a single ASV (*Chroococcidiopsis*) was discriminatory for habitat disturbance indicates that disturbance either drives stochastic, unpredictable changes in taxa abundance across sites, as has previously been demonstrated [33], or is not driving large enough shifts in taxa abundance to be statistically significant. The latter scenario of minimal impact of habitat disturbance on the host skin microbiome may indicate that *D. dhufarensis* has a degree of resilience to microbiome perturbation. The higher capacity to cope with anthropogenic disturbances has been observed in the closely related Asian toad (*Duttaphrynus melanostictus*), collected from areas that are impacted by human activity in Madagascar, and may contribute to the success of this species in invasively colonising novel habitats [84].

Our network analyses demonstrated comparable network properties across disturbed and undisturbed sites, with the majority of strong edge interactions (*ρ* > 0.7 or *ρ* < − 0.7) detected across networks being positive. The number of common edges between sites was relatively small compared to the number of common taxa (or nodes). This indicates that although amphibians from different sites may share common bacterial taxa, the patterns of co-occurrence between these taxa differ greatly, and host microbial community dynamics are therefore likely to vary. This finding suggests that factors unique to individual sites (e.g. local environment) are important in shaping skin bacterial community composition and assembly [85], with habitat disturbance not driving clear or predictable shifts in patterns of taxa co-occurrence.

Our finding that the microbiome is strongly structured by host life stage, with increased alpha diversity in adults compared to larvae, along with significant differences in beta diversity, is consistent with results of prior studies [16, 86–88]. The small percentage of ASVs (9%) with a relative abundance > 0.01% present in the post-metamorphic microbiome that were also found in larvae, large number of differentially abundant taxa, and few common bacterial co-occurrences further demonstrates significant restructuring of the skin microbiome from larvae to adults, in line with findings from other amphibian species [16, 87–90]. Although larval and adult networks differed substantially in taxa composition, the broad similarities in network topologies indicate that amphibian skin supports similarly complex bacterial ecosystems across host life stages. These changes in community composition and bacterial co-occurrence likely reflect the extensive physiological reprogramming that occur during amphibian metamorphosis [91, 92] and the resulting shift in microbial niche that arises.

Overall, our findings support those of prior studies, as well as generate novel insight into the factors that shape the amphibian skin microbiome. As one of only two amphibian species endemic to Oman, understanding the link between host environment, pathogen presence, dermal microbiome, and host health is crucial to help maintain the long-term viability of the Dhofar toad. What is more, as a desert/semi-arid living species, *D*. *dhufarensis* may prove valuable as a comparative model of skin microbial community dynamics with hosts from other climates. Finally, our work hints at the potential value of the microbiome as a metric for habitat disturbance, with future work necessary to determine whether signatures of ecosystem distress can be detected across greater spatial scales.

## Supplementary Information

Below is the link to the electronic supplementary material.

Supplementary file1 (XLSX 31.4 KB)

Supplementary file2 (PDF 10.8 MB)

## Data Availability

16S rRNA gene sequence data have been deposited on the BioProject database under accession code PRJNA899512. All other data and code are available upon reasonable request from the authors.
