# Supplementary material for: Environmental and Anthropogenic Factors Shape the Skin Bacterial Communities of a Semi-Arid Amphibian Species
Source: Microb Ecol. 2022 Nov 29;86(2):1393–404. doi: 10.1007/s00248-022-02130-5 (PMC10335963; doi:10.1007/s00248-022-02130-5)
Supplement: Supplementary file 2 — Supplementary file2 (PDF 10.8 MB) [file 248_2022_2130_MOESM2_ESM.pdf]

<u>Supplementary Figures</u>

SI Fig 1. PCA plot of ranavirus presence/absence among post-metamorphic animals.

SI Fig 2. Boxplot of CLR transformed abundance of ASV138 *Chroococcidiopsis*.

SI Fig 3. ASVs common among environment and host samples for each population.

SI Fig 4. ASVs common among post-metamorphic animals from each population.

SI Fig 5. (**A-F**) Bacterial networks for each adult population. Hub taxa are highlighted by yellow node perimeters. Positive and negative associations (edges) between taxa are coloured grey and red respectively. (**G**) Node frequencies and taxonomic assignments.

SI Fig 6. Shared Hub taxa identified from network analysis among post-metamorphic populations.

SI Fig 7. Shared edges identified from network analysis among post-metamorphic populations.

SI Fig 8. A) Larval and **B)** Adult networks for Wadi Na'ar. Hub taxa are highlighted by yellow node perimeters. Positive and negative associations (edges) between taxa are coloured grey and red respectively.

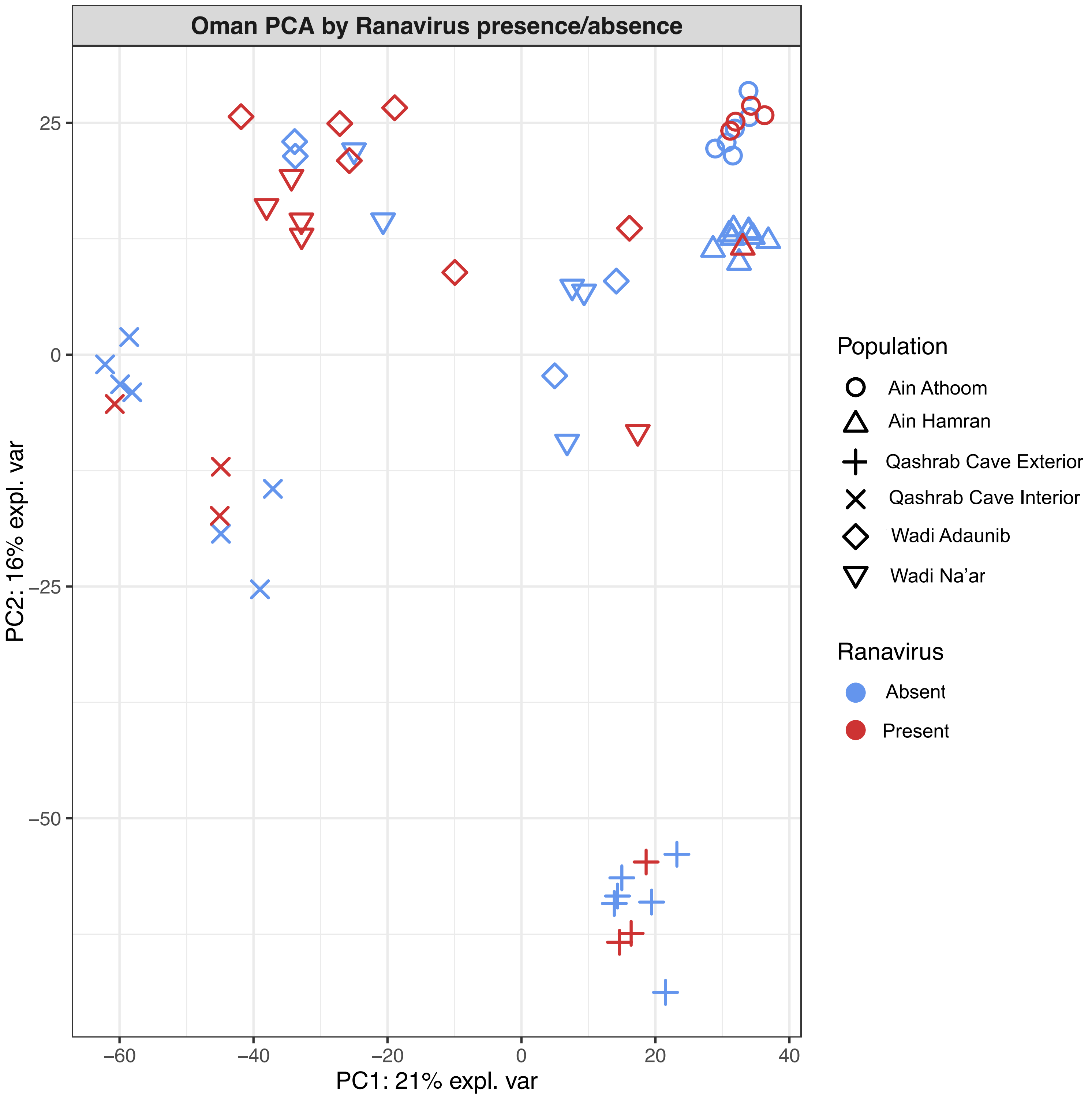
Oman PCA by Ranavirus presence/absence
PC1: 21% expl. var
PC2: 16% expl. var
−60
−40
−20
0
20
40
25
0
−25
−50
Population
Ain Athoom
Ain Hamran
Qashrab Cave Exterior
Qashrab Cave Interior
Wadi Adaunib
Wadi Na'ar
Ranavirus
Absent
Present

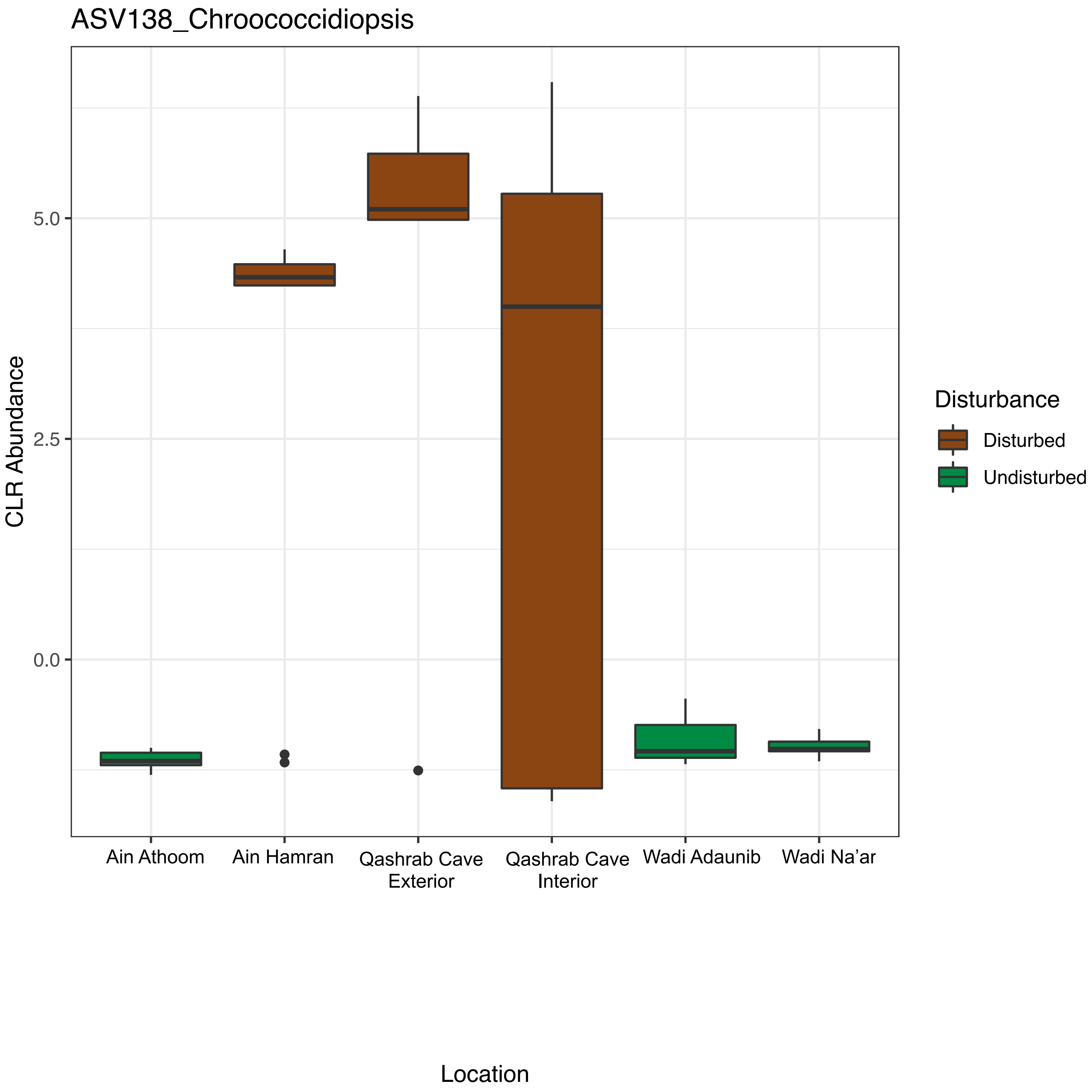
ASV138_Chroococcidiopsis
CLR Abundance
5.0
2.5
0.0
Ain Athoom
Ain Hamran
Qashrab Cave Exterior
Qashrab Cave Interior
Wadi Adaunib
Wadi Na'ar
Location
Disturbance
Disturbed
Undisturbed

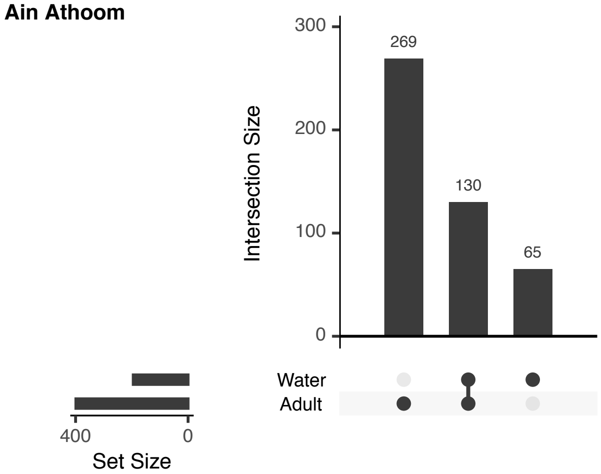
Ain Athoom
Intersection Size
0
100
200
300
269
130
65
Water
Adult
Set Size
400
0

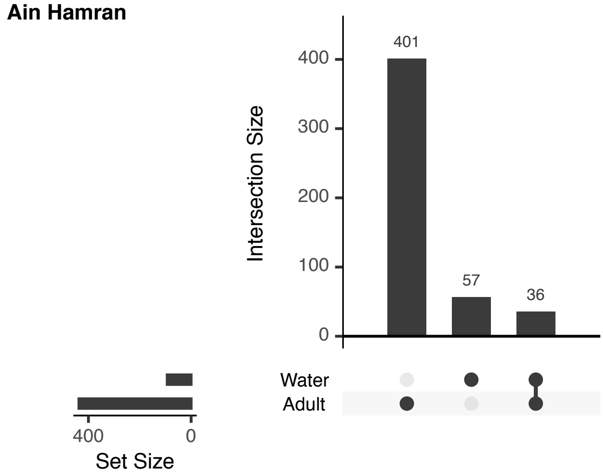
Ain Hamran
Intersection Size
0
100
200
300
400
401
57
36
Water
Adult
Set Size
400
0

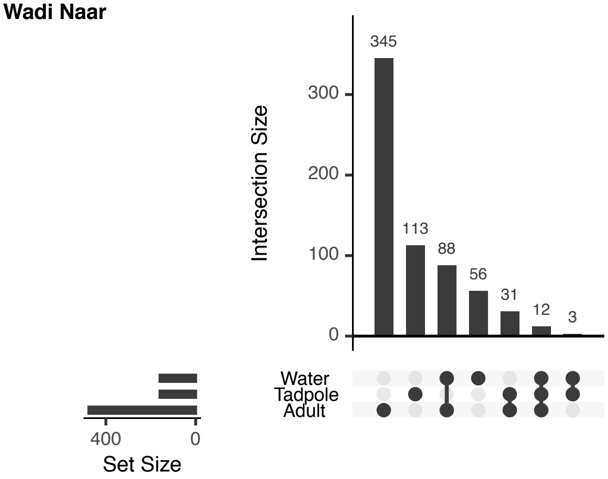
Wadi Naar
Intersection Size
0
100
200
300
345
113
88
56
31
12
3
Water
Tadpole
Adult
Set Size
400
0

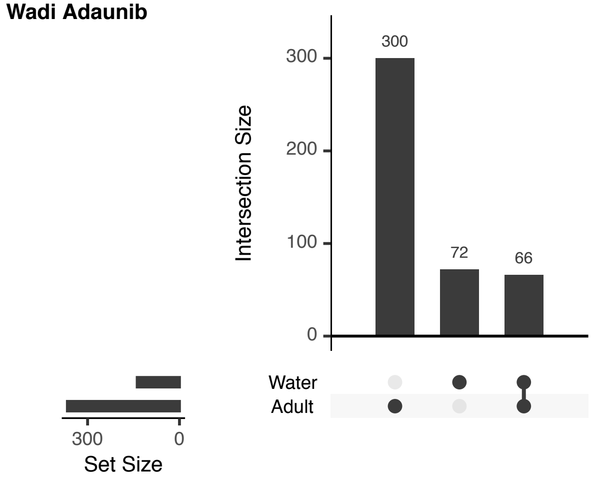
Wadi Adaunib
Intersection Size
0
100
200
300
300
72
66
Water
Adult
Set Size
300
0

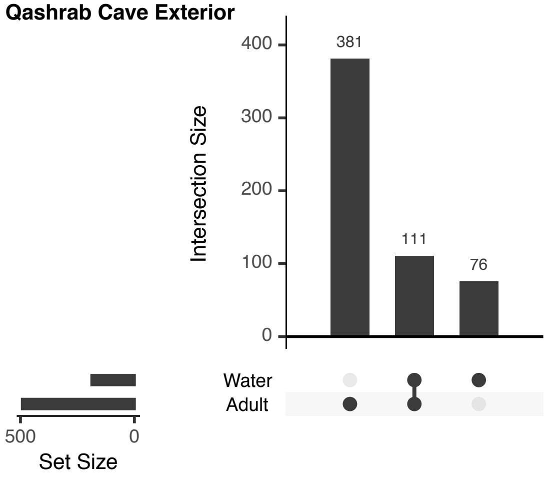
Qashrab Cave Exterior
Intersection Size
0
100
200
300
400
381
111
76
Water
Adult
Set Size
500
0

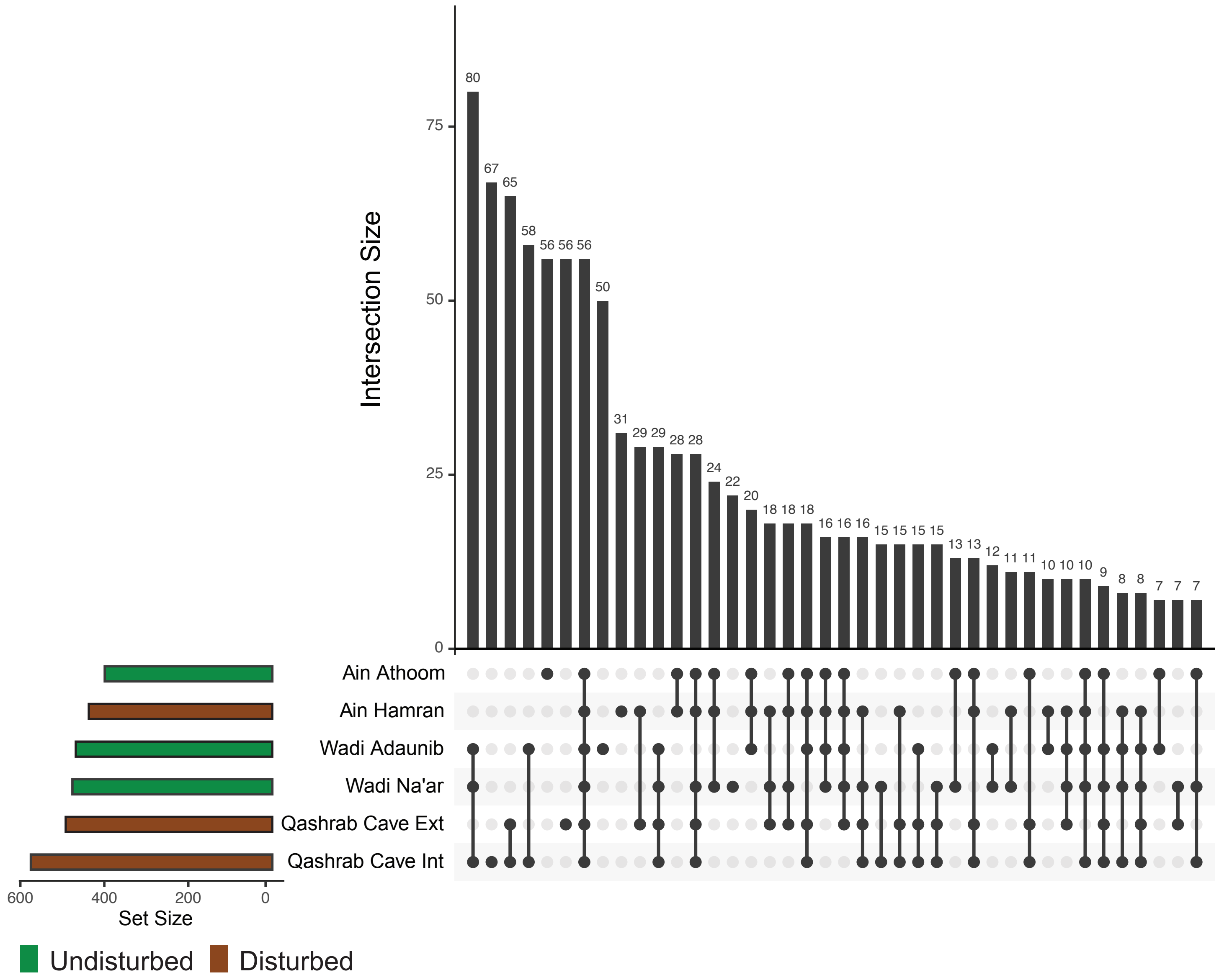
Intersection Size
80
67
65
58
56 56 56
50
31
29 29
28 28
24
22
20
18 18 18
16 16 16
15 15 15 15
13 13
12
11 11
10 10 10
9
8 8
7 7 7
75
50
25
0
Ain Athoom
Ain Hamran
Wadi Adaunib
Wadi Na'ar
Qashrab Cave Ext
Qashrab Cave Int
600
400
200
0
Set Size
Undisturbed
Disturbed

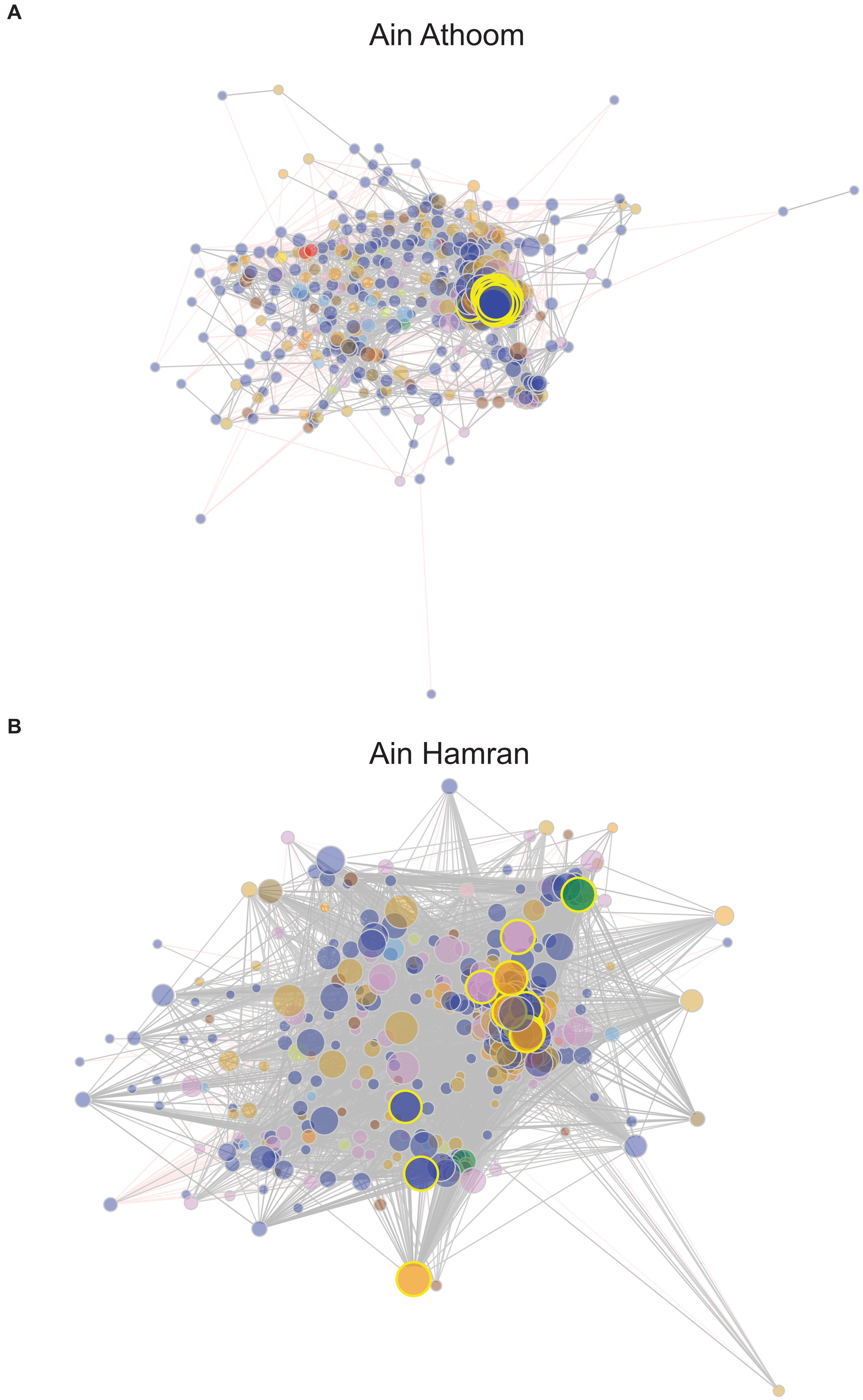
A
Ain Athoom
B
Ain Hamran

C

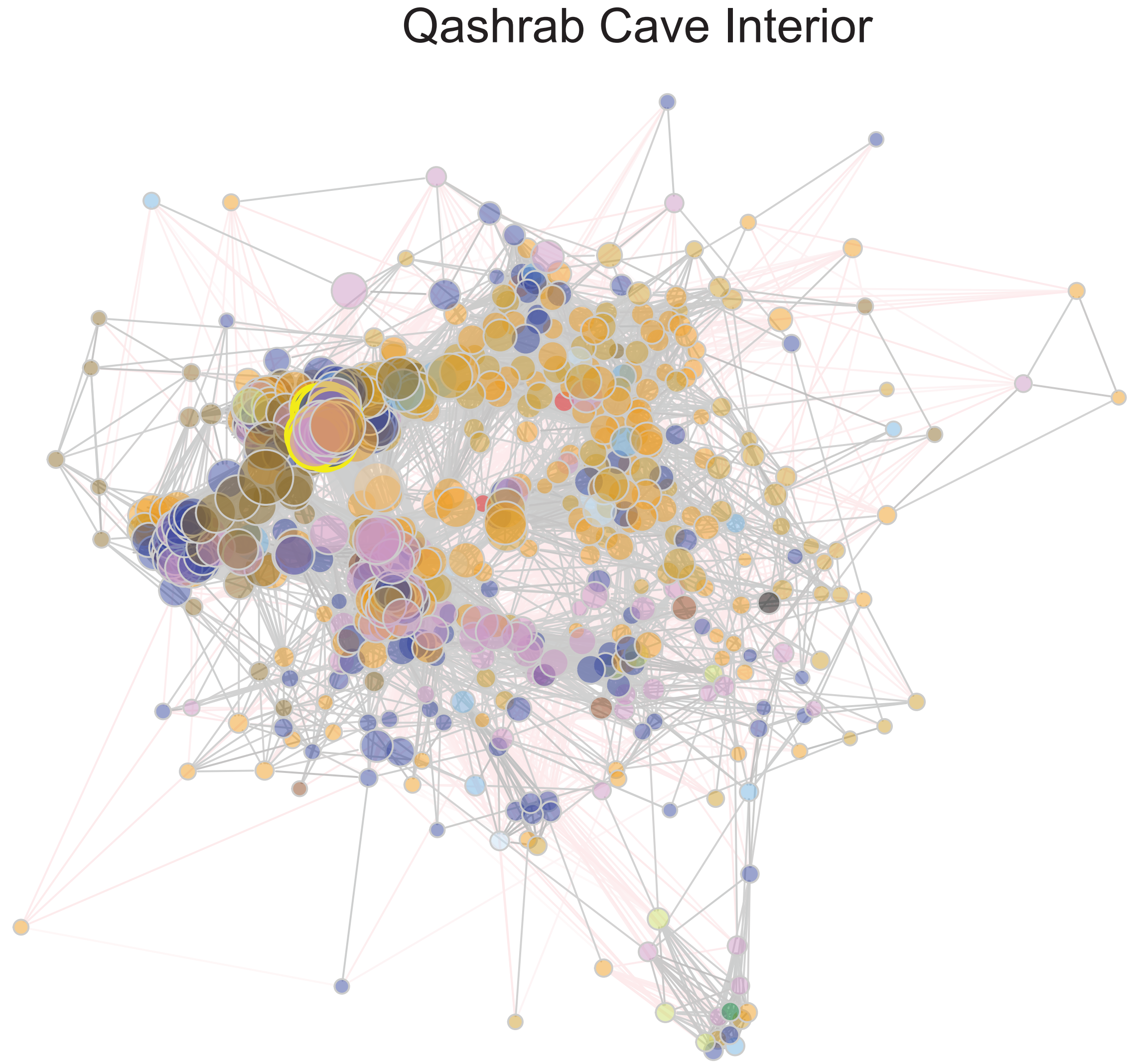

D

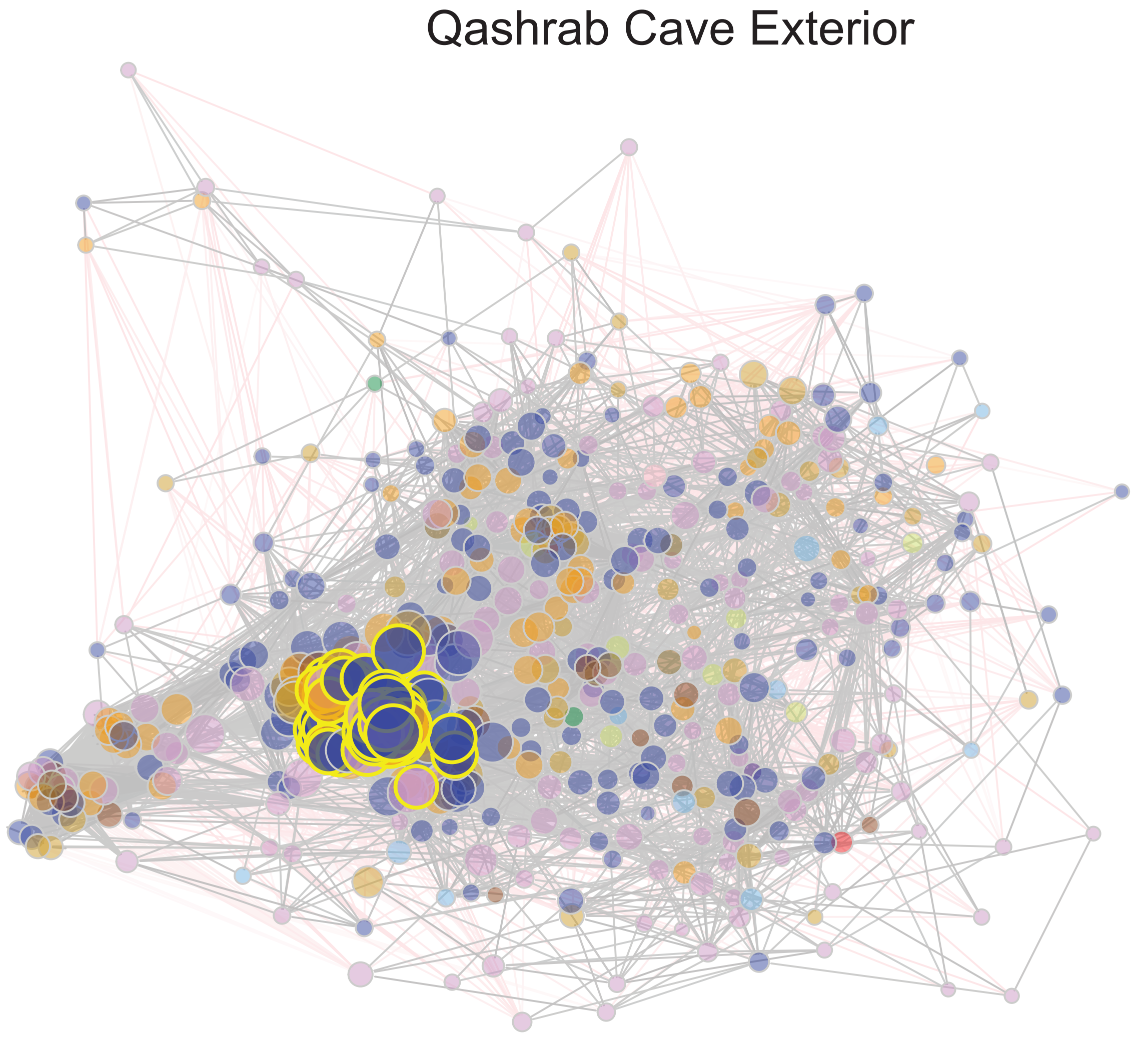

E

Wadi Na’ar

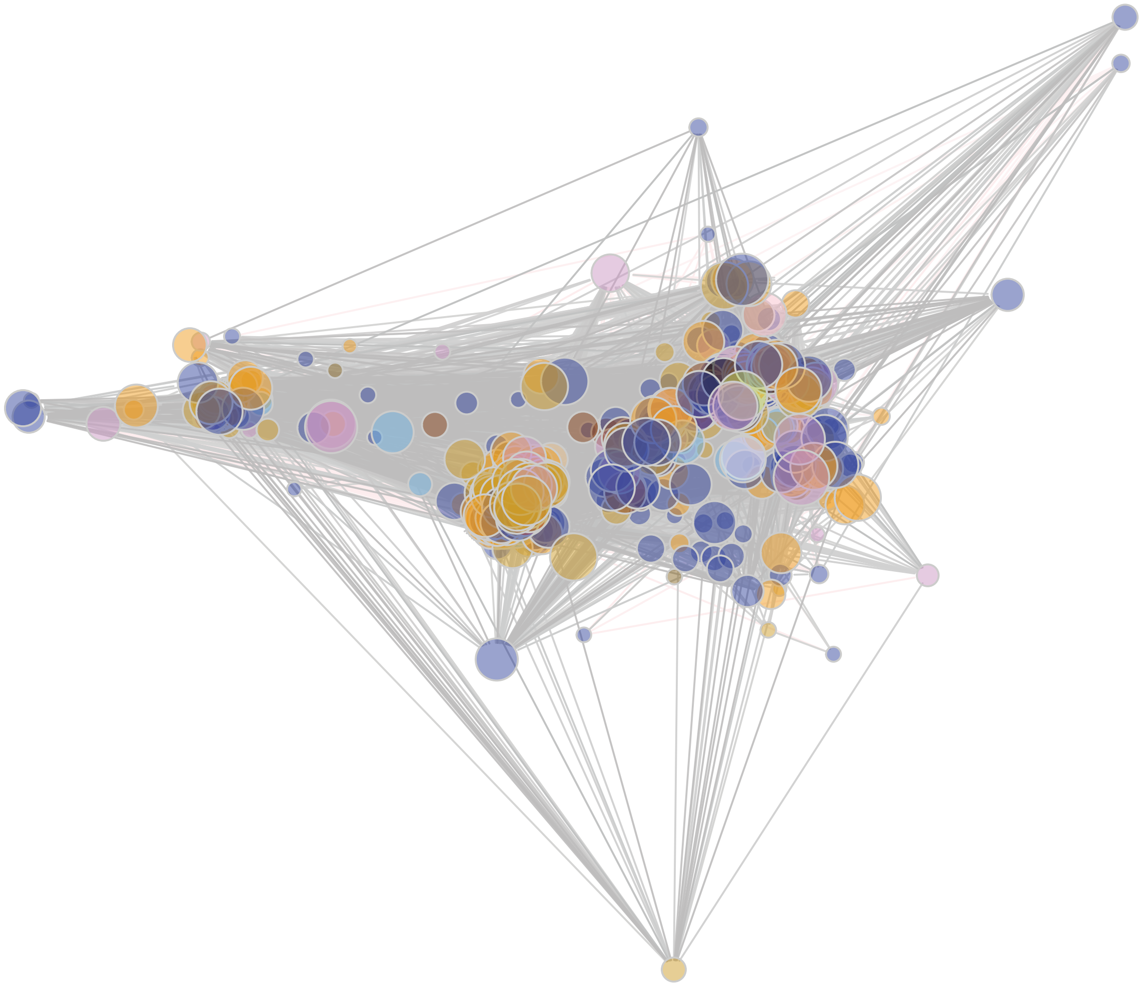

F

Wadi Adaunib

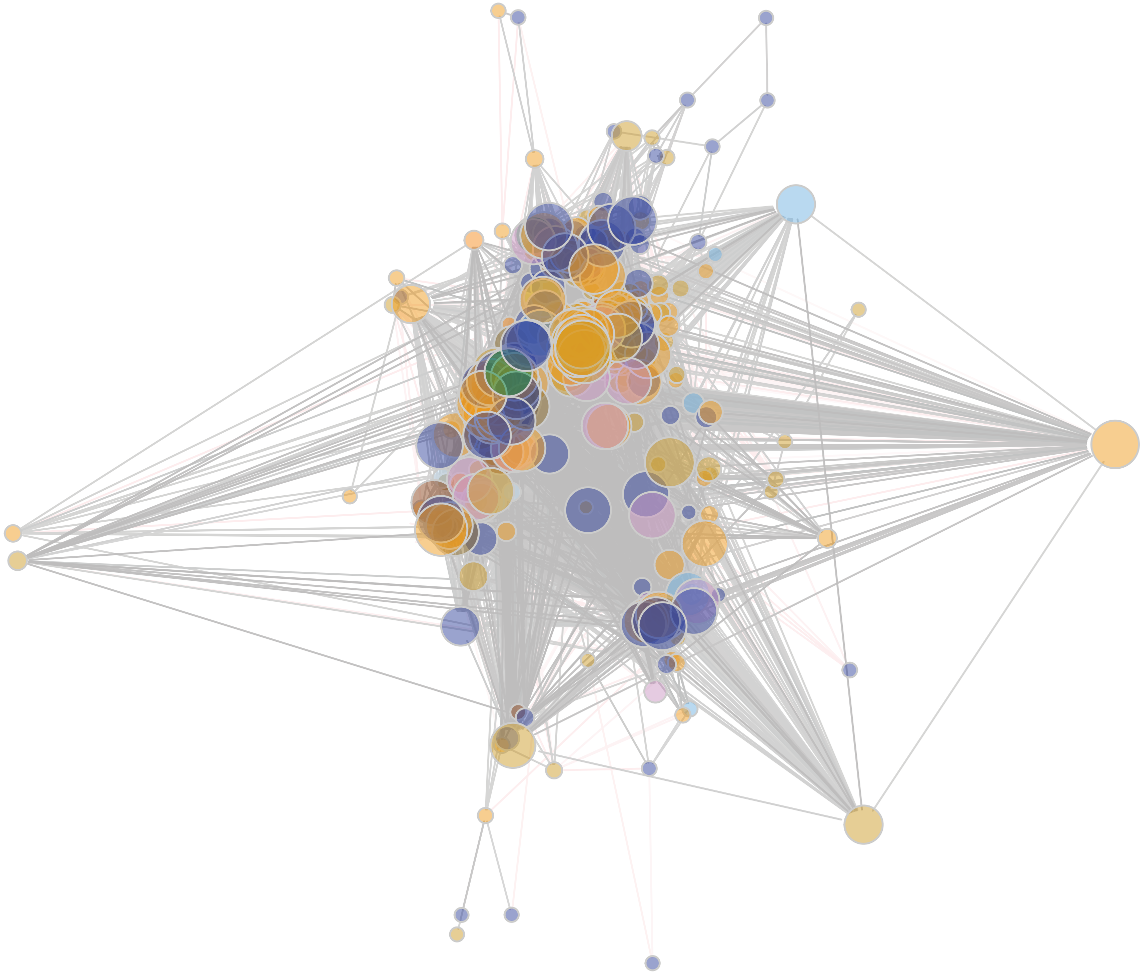

G

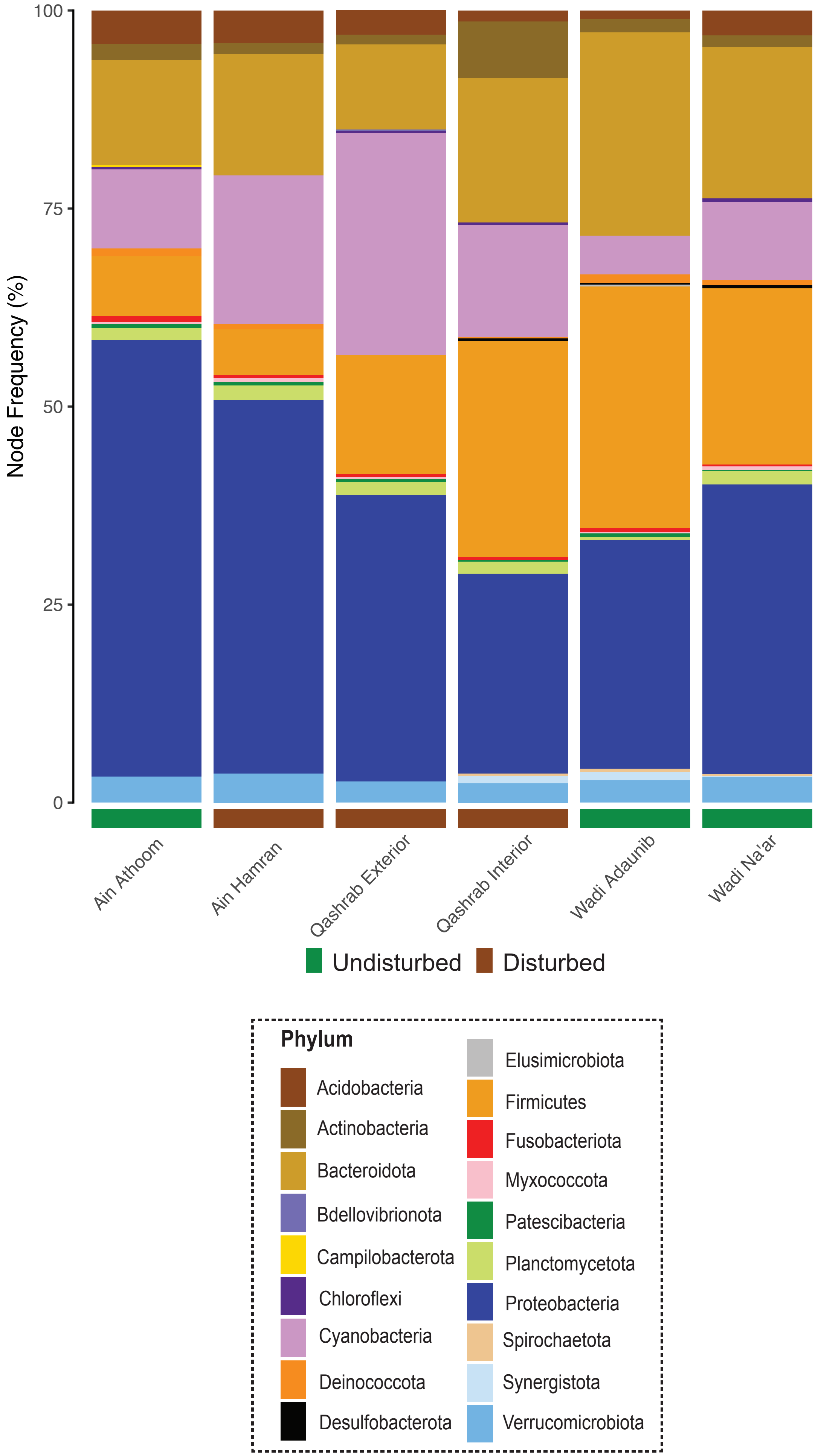

Node Frequency (%)
100
75
50
25
0
Ain Athoom
Ain Hamran
Qashrab Exterior
Qashrab Interior
Wadi Adaunib
Wadi Na'ar
Undisturbed
Disturbed
Phylum
Acidobacteria
Actinobacteria
Bacteroidota
Bdellovibrionota
Campilobacterota
Chloroflexi
Cyanobacteria
Deinococcota
Desulfobacterota
Elusimicrobiota
Firmicutes
Fusobacteriota
Myxococcota
Patescibacteria
Planctomycetota
Proteobacteria
Spirochaetota
Synergistota
Verrucomicrobiota

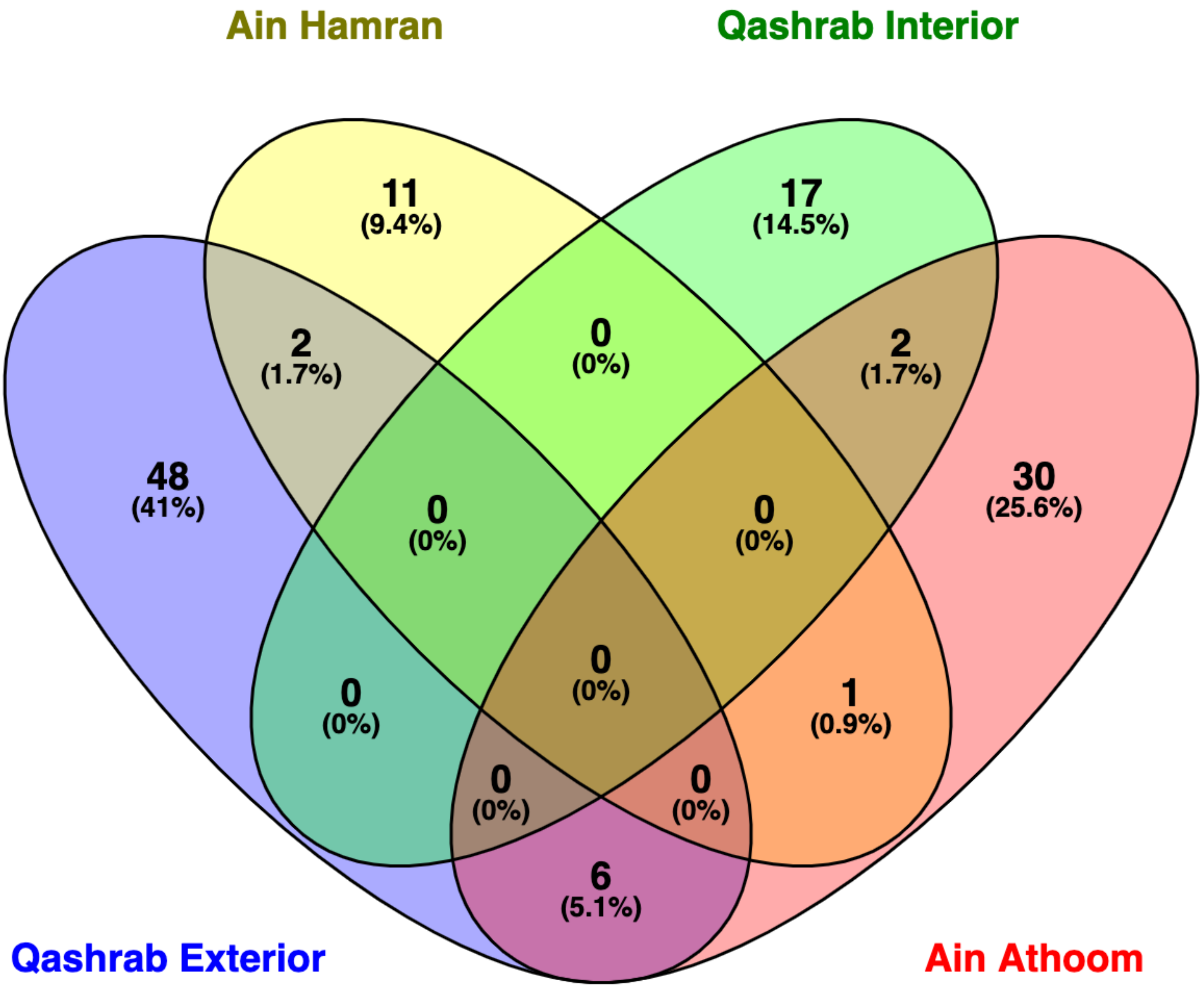
Ain Hamran
Qashrab Interior
Qashrab Exterior
Ain Athoom
11
(9.4%)
17
(14.5%)
2
(1.7%)
0
(0%)
2
(1.7%)
48
(41%)
0
(0%)
0
(0%)
30
(25.6%)
0
(0%)
0
(0%)
1
(0.9%)
0
(0%)
0
(0%)
6
(5.1%)

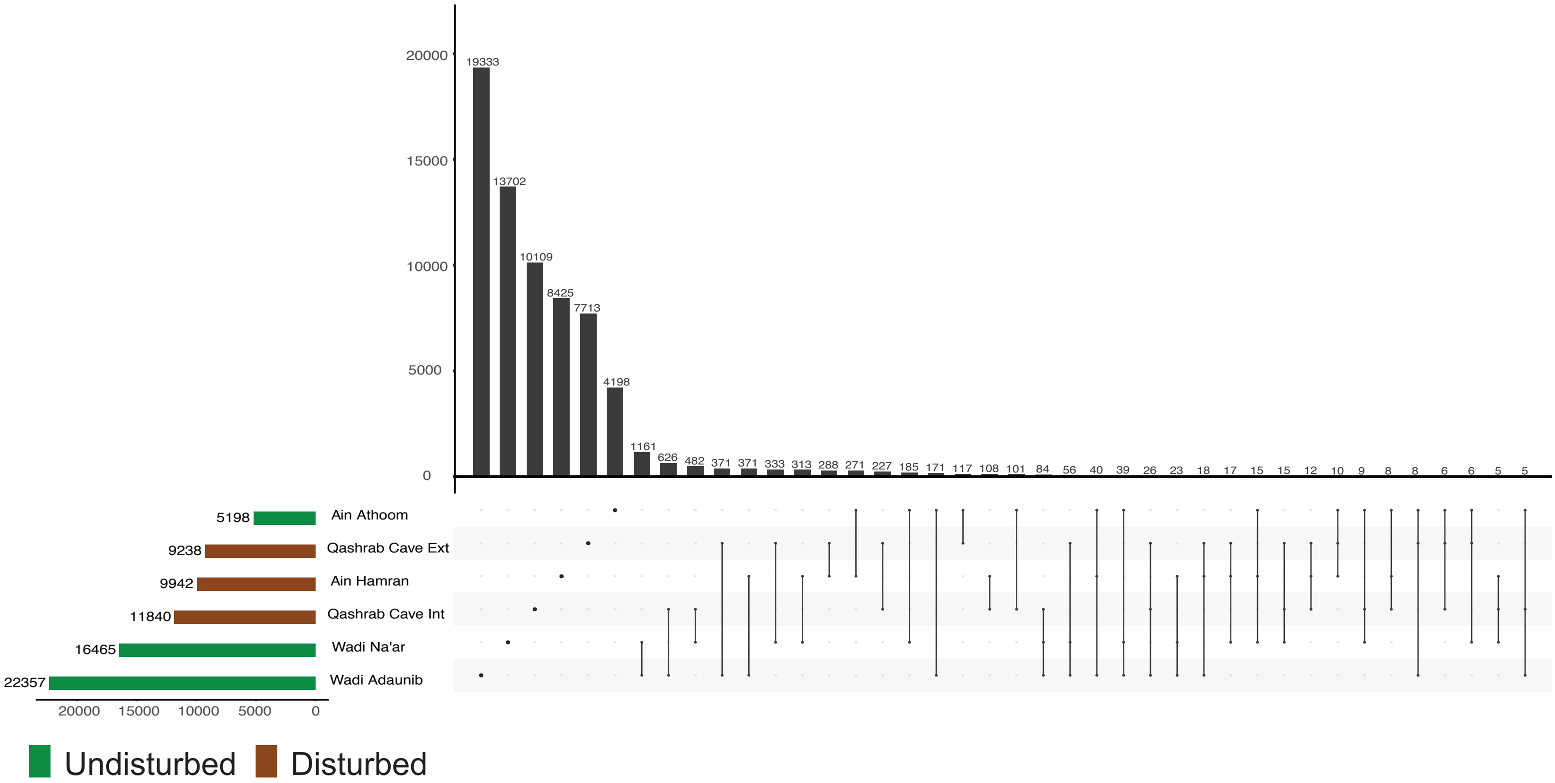

20000
15000
10000
5000
0
19333
13702
10109
8425
7713
4198
1161
626
482
371
371
333
313
288
271
227
185
171
117
108
101
84
56
40
39
26
23
18
17
15
15
12
10
9
8
8
6
6
5
5
5198
Ain Athoom
9238
Qashrab Cave Ext
9942
Ain Hamran
11840
Qashrab Cave Int
16465
Wadi Na'ar
22357
Wadi Adaunib
20000
15000
10000
5000
0
Undisturbed
Disturbed

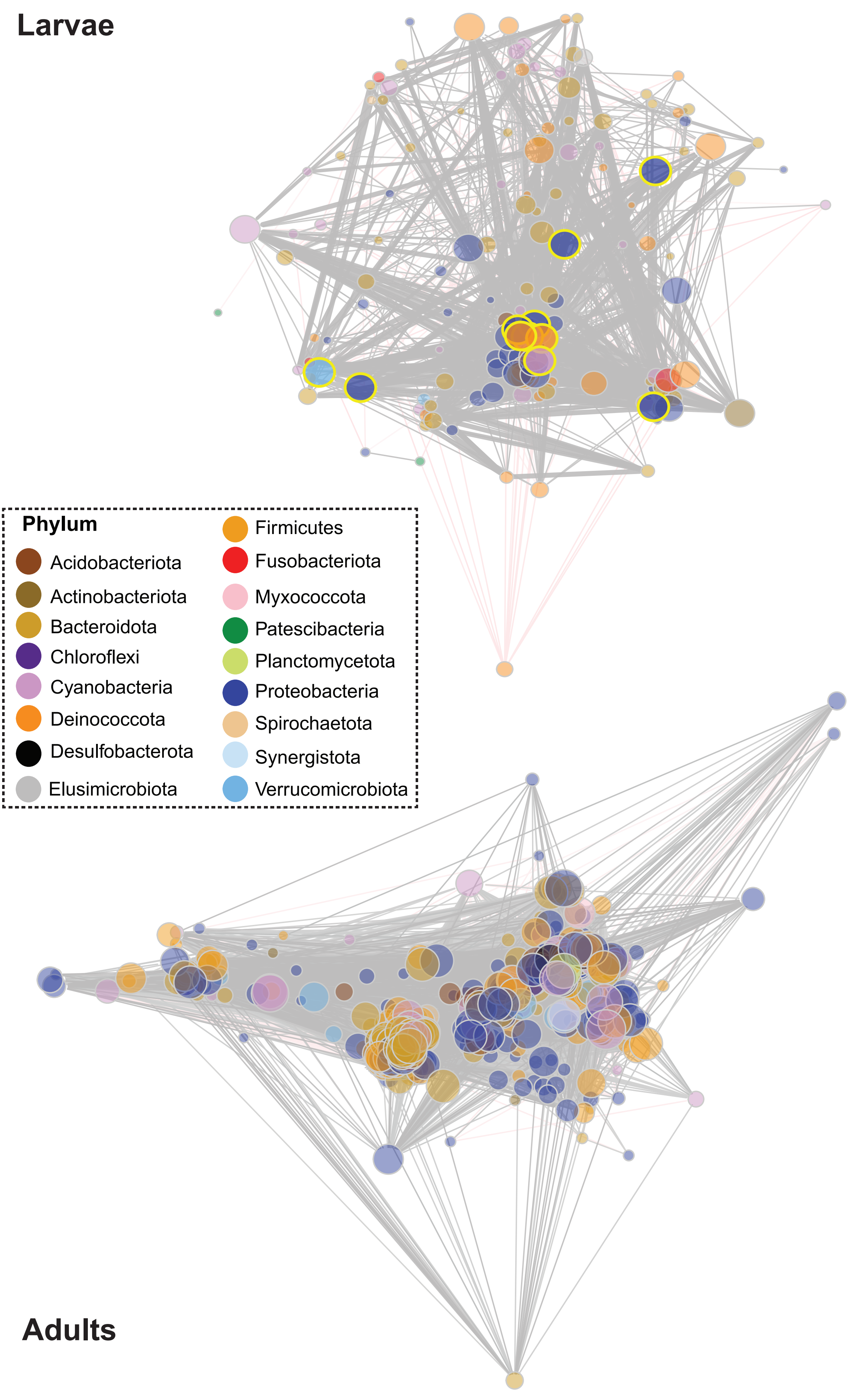

Larvae
Phylum
Acidobacteriota
Actinobacteriota
Bacteroidota
Chloroflexi
Cyanobacteria
Deinococcota
Desulfobacterota
Elusimicrobiota
Firmicutes
Fusobacteriota
Myxococcota
Patescibacteria
Planctomycetota
Proteobacteria
Spirochaetota
Synergistota
Verrucomicrobiota
Adults
